# Laccases: Production, Expression Regulation, and Applications in Pharmaceutical Biodegradation

**DOI:** 10.3389/fmicb.2017.00832

**Published:** 2017-05-16

**Authors:** Jie Yang, Wenjuan Li, Tzi Bun Ng, Xiangzhen Deng, Juan Lin, Xiuyun Ye

**Affiliations:** ^1^Fujian Key Laboratory of Marine Enzyme Engineering, Fuzhou UniversityFujian, China; ^2^Faculty of Medicine, School of Biomedical Sciences, The Chinese University of Hong KongShatin, Hong Kong

**Keywords:** laccase, production, expression regulation, bioremediation, PPCPs, antibiotics

## Abstract

Laccases are a family of copper-containing oxidases with important applications in bioremediation and other various industrial and biotechnological areas. There have been over two dozen reviews on laccases since 2010 covering various aspects of this group of versatile enzymes, from their occurrence, biochemical properties, and expression to immobilization and applications. This review is not intended to be all-encompassing; instead, we highlighted some of the latest developments in basic and applied laccase research with an emphasis on laccase-mediated bioremediation of pharmaceuticals, especially antibiotics. Pharmaceuticals are a broad class of emerging organic contaminants that are recalcitrant and prevalent. The recent surge in the relevant literature justifies a short review on the topic. Since low laccase yields in natural and genetically modified hosts constitute a bottleneck to industrial-scale applications, we also accentuated a genus of laccase-producing white-rot fungi, *Cerrena*, and included a discussion with regards to regulation of laccase expression.

## Introduction

Laccases (EC 1.10.3.2) are a family of copper-containing oxidases found in a variety of bacteria, fungi, insects, and plants (Forootanfar and Faramarzi, 2015). The four copper atoms of a typical laccase molecule are divided into Type 1 (T1), Type 2 (T2), and binuclear Type 3 (T3) Cu sites based on unique spectroscopic features. In the resting enzyme, the four copper ions are in the +2 oxidation state. The T1 and T3 coppers are characterized by absorption at ~600 and 330 nm, respectively, whereas the T2 site lacks strong absorption features. Substrate oxidation occurs at the T1, and electrons are transferred to the T2/T3 trinuclear copper cluster (TNC), where molecular oxygen is reduced to water (Wong, 2009; Jones and Solomon, 2015). Redox potentials of the T1 sites in laccases range from 0.4 to 0.8 V; plant and bacterial laccases (e.g., 0.43 and 0.46 V for *Rhus vernicifera* and wild-type *Bacillus subtilis* CotA laccases, respectively) typically have potentials on the low end of this range, whereas fungal laccases have higher redox potentials (0.47–0.79 V) (Forootanfar and Faramarzi, 2015; Jones and Solomon, 2015; Mate and Alcalde, 2015; Pogni et al., 2015). With one-electron oxidation and radical formation, laccases catalyze oxidative coupling or bond cleavage of target compounds (Jeon and Chang, 2013).

Laccases have diverse substrate spectra, which overlap with those of tyrosinase and bilirubin oxidase (Baldrian, 2006; Reiss et al., 2013). They can oxidize a wide range of compounds, such as mono-, di-, poly-, and methoxy-phenols, aromatic and aliphatic amines, hydroxyindoles, benzenethiols, carbohydrates, and inorganic/organic metal compounds (Giardina et al., 2010; Jeon et al., 2012; Karaki et al., 2016). ABTS is the most popular substrate in laccase activity assays, and 2,6-dimethoxyphenol (2,6-DMP), catechol, guaiacol, and syringaldazine are also commonly used. The scope of laccase substrates can be further broadened with the help of redox mediators from natural and synthetic sources, i.e., suitable laccase substrates that can serve as diffusible electron shuttles between enzymes and other compounds (Morozova et al., 2007; Cañas and Camarero, 2010).

Since laccases have wide substrate ranges and use only oxygen as the final electron receptor, they have widespread applications in various industries, such as textile, food, biofuel, organic synthesis, bioremediation, paper and pulp, pharmaceutical, and cosmetic industries (Arora and Sharma, 2010; Majeau et al., 2010; Osma et al., 2010; Kudanga et al., 2011; Jeon et al., 2012; Betancor et al., 2013; Kudanga and Roes-Hill, 2014; Mogharabi and Faramarzi, 2014; Viswanath et al., 2014; Pezzella et al., 2015; Mate and Alcalde, 2016; Senthivelan et al., 2016; Sitarz et al., 2016; Upadhyay et al., 2016). In fact, a few laccase products are already commercially available for food, paper, textile, and other industries (Osma et al., 2010; Rodríguez-Couto, 2012). Laccase-based biocatalysts fit well with the development of industries that are efficient, sustainable, and environment-friendly.

Nonetheless, large-scale applications of laccases are limited by the economy and efficiency of the enzymes (Osma et al., 2010; Strong and Claus, 2011; Singh G. et al., 2015). Efforts have been made to produce large amounts of laccases at lower costs with the use of recombinant organisms or screening for natural hypersecretory strains. Enzyme activity and stability can be improved through immobilization and protein engineering (Pezzella et al., 2015; Upadhyay et al., 2016). The present article is not intended to be a comprehensive review on laccases, instead, it highlights the latest developments in laccase production and applications in bioremediation, especially degradation of emerging micropollutants including antibiotics.

## Natural laccase producers

### Laccase-producing fungi

Although, the first discovered laccase came from the exudates of the plant *R. vernicifera*, laccases of fungal origins have been the most intensively studied. Fungal laccases are implicated in both intra- and extra-cellular physiological processes including delignification, morphogenesis, pigmentation, and pathogenesis (Arora and Sharma, 2010; Kües and Rühl, 2011; Forootanfar and Faramarzi, 2015).

Among fungi, ascomycetes, basidiomycetes, and deuteromycetes can produce laccases, and white-rot basidiomycetes are the most efficient lignin degraders and laccase producers (Rodríguez-Couto and Toca-Herrera, 2007; Arora and Sharma, 2010). Laccases are secreted by white-rot fungi along with other ligninolytic enzymes including manganese peroxidase, lignin peroxidase, and versatile peroxidase, although the specific types of enzymes secreted may differ with the fungus (Wong, 2009; Arora and Sharma, 2010).

*Pleurotus ostreatus* and *Trametes versicolor* can be regarded as the model organisms in basic and applied laccase research. Other well-known laccase-producing basidiomycetes include *Agaricus bisporus, Cerrena unicolor, Coprinopsis cinerea, Coriolopsis gallica, Cryptococcus neoformans, Cyathus bulleri, Fomes fomentarius, Ganoderma lucidum, Panus rudis, Phlebia radiata, Polyporus brumalis, Pycnoporus cinnabarinus, Pycnoporus sanguineus, Rigidoporus microporus, Schizophyllum commune*, as well as various *Pleurotus* (e.g., *P. eryngii, P. florida, P. pulmonarius*, and *P. sajor-caju*) and *Trametes* (e.g., *T. hirsuta, T. pubescens, T. trogii*, and *T. villosa*) species (Baldrian, 2006; Arora and Sharma, 2010; Forootanfar and Faramarzi, 2015).

Efforts are still being made to screen naturally-occurring laccase producers with desired laccase yields and properties (Chen et al., 2012; Si et al., 2013; Fang Z. et al., 2015; Iracheta-Cárdenas et al., 2016; Kandasamy et al., 2016; Olajuyigbe and Fatokun, 2017). Laccase yields are variable depending on the species and strain, but most naturally-occurring species appear to be poor laccase producers. However, screening and selection of promising laccase producers from nature, followed by optimization of culture conditions, still constitute a viable and effective approach to obtain organisms with tremendous laccase synthesis ability (Elisashvili and Kachlishvili, 2009). The genus *Cerrena* with high laccase yields and application potentials deserves attention, and the properties of its laccase can be even more desirable compared to the commercial ones (Chen et al., 2012). However, *Cerrena* species are relatively less studied, especially compared with *Trametes* species. *C. unicolor*, a medicinal mushroom with antitumor and other activities (Mizerska-Dudka et al., 2015; Matuszewska et al., 2016), has been reported as a constitutive laccase producer (Al-Adhami et al., 2002); indeed, for many reported *Cerrena* strains, an organic inducer is not necessary, but copper ions are beneficial for laccase production. On the contrary, there are also some *Cerrena* strains that respond to lignocellulosic substrates or aromatic compounds, corroborating that enzyme production varies with the strain and should be characterized for each potentially valuable strain. Laccase production by reported *Cerrena* species is summarized in Table 1, which is comparable to that by *Trametes* species (Majeau et al., 2010) or *G. lucidum* (Postemsky et al., 2017).

**Table 1:** **Summary of laccase production by the genus *Cerrena***.

**Strain**	**Yield (U/mL)**	**Cycle (d)**	**Carbon and nitrogen sources**	**Inducer**	**References**
*Cerrena unicolor* C-139	0.5^a^^,^^b^	7	Glucose 20 g/L, _*L*_-asparagine 2.5 g/L	Cu^2+^ (8 μM)	Al-Adhami et al., 2002
*Cerrena unicolor* C-139	3.9^b^^,^^c^^,^^d^	8	Lindeberg-Holm medium (glucose 10 g/L, _*L*_-asparagine 1.5 g/L)	Cu^2+^ (10 μM) added at 3 and 6 d	Janusz et al., 2007; Rogalski and Janusz, 2010
*Cerrena unicolor* C-139	250^a^/450^a^	7/14	Glucose 10 g/L, wheat bran (40 g/L), bacto peptone 2 g/L, yeast extract 2 g/L	Cu^2+^ (1 mM), wheat bran	Songulashvili et al., 2012
*Cerrena unicolor* C-139	416.4^e^	12	Glucose 5.5 g/L, wheat bran 40 g/L, peptone 2g/L, yeast extract 2g/L	Cu^2+^ (1 mM), wheat bran	Songulashvili et al., 2015
*C. unicolor* 137	18.7^a^	12	50% eco-tomato juice	–	Michniewicz et al., 2006
*C. unicolor* 137	4^a^	12	Modified Kirk medium (glucose 13 g/L, di-ammonium tartrate 0.5 g/L, yeast extract 0.25 g/L)	Cu^2+^ (50 μM)	Michniewicz et al., 2006
*C. unicolor* MTCC 5159	85.8^a^	12	B&K medium (glucose 10 g/L, peptone 2 g/L, yeast extract 1 g/L)	Textile effluent (1%)	D'Souza et al., 2006
*C. unicolor* VKMF-3196	15^a^	8	Kirk medium with high nitrogen (0.9 g/L α-asparagine and NH_4_NO_3_)	Cu^2+^ (0.1 mM)	Lisova et al., 2010
*C. unicolor* IBB 300	151.6^a^	14	Wheat bran 40 g/L	Wheat bran	Elisashvili and Kachlishvili, 2009
*C. unicolor* IBB 300	165^a^	NA	Ethanol production residue 40 g/L, ammonium tartrate 2 g/L, yeast extract 3 g/L	2,4,6-trinitrotoluene (0.5 mM)	Elisashvili and Kachlishvili, 2009; Elisashvili et al., 2010
*C. unicolor* IBB 300	20^a^	4	Ammonium tartrate 2 g/L, yeast extract 3 g/L, mannitol 10	Cu^2+^ (0.1 mM), 2,4,6-trinitrotoluene (0.3 mM)	Elisashvili and Kachlishvili, 2009; Elisashvili et al., 2010
*Cerrena* sp. WR1	202^f^	13	2.4% potato dextrose broth (Difco, BD), 5% soytone	Cu^2+^ (0.4 mM), 2,5-xylidine (2 mM)	Chen et al., 2012
*Cerrena* sp. Ra	5^a^^,^^g^	6	Glucose 10 g/L, polypeptone 5 g/L, yeast extract 1 g/L	–	Hibi et al., 2012
*Cerrena* sp. HYB07	280^a^	5	Maltodextrin 60 g/L, peptone 10 g/L	Cu^2+^ (0.25 mM)	Yang et al., 2016a
*C. unicolor* GSM-01	2,800^a^^,^^h^	8	Potato dextrose medium	Cu^2+^ (1 mM)	Wang et al., 2017

a *Fermentation was carried out in a shake flask*.

b *Enzyme activity was assayed with syringaldazine as the substrate*.

c *Fermentation was carried out in a 2.5-L fermenter*.

d *Enzyme activity was converted from nkat to U by dividing by 16.67*.

e *Fermentation was carried out in a 120-L fermenter*.

f *Fermentation was carried out in a 5-L fermenter*.

g *Enzyme activity was assayed with Remazol Brilliant Blue R as the substrate*.

h *Enzyme activity was assayed with ABTS as the substrate at 405 nm. Unless otherwise mentioned, enzyme activity was assayed with ABTS as the substrate at 420 nm. NA, not available*.

Fungal laccases exist in gene families, and reported basidiomycete laccase gene families contain 5–17 members (Table 2). Laccase isozymes are compared with respect to sequences, phylogenetic relationship, catalytic properties, and expression regulation. Sequence and evolutionary relationship examinations indicate that modern laccase gene families are derived from duplication-divergence events of a small set of ancestral enzymes (Valderrama et al., 2003; Kilaru et al., 2006a; Courty et al., 2009; Kües and Rühl, 2011; Bao et al., 2013; Wang W. et al., 2015). During natural evolution, the laccase paralogs may diversify in their functions, which is supported by numerous biochemical and expression characterization data (Hoegger et al., 2004, 2006; Pezzella et al., 2013; Fan et al., 2014; Yang et al., 2016b). Expression patterns provide valuable information for deducing the physiological roles played by the laccase isoforms. For example, Lcc5 in *Auricularia auricula-judae* is implicated in the sexual reproduction stage (Fan et al., 2014), LACC10 of *P. ostreatus* seems to function during vegetative growth (Pezzella et al., 2013), and Lcc3 is possibly involved in stipe elongation of *Volvariella volvacea* (Lu et al., 2015). Transcriptomic analysis of *Flammulina velutipes* implies that laccase isozymes are involved in growth and development, such as lignin bioconversion, stipe elongation and pileus formation (Wang W. et al., 2015). Sometimes, functional redundancy among laccase isozymes is suggested (Sakamoto et al., 2015; Wang W. et al., 2015). Furthermore, evidence for *in vivo* laccase function has been provided by genetic experiments. A siRNA knockdown study demonstrates that Lcc2 in *A. bisporus* contributes to toxin metabolism and defense against green mold disease (Sjaarda et al., 2015). On the other hand, overexpression of *Hypsizygus marmoreus* Lcc1 facilitates mycelial growth and fruiting body initiation (Zhang et al., 2015). Indeed, laccase has been developed as a novel screening marker in mushroom breeding (Sun et al., 2014). The levels of secreted laccase activity in edible mushrooms and their growing cycles are closely related, and short growing cycles are accompanied by high laccase activity (Sun et al., 2011).

**Table 2:** **Basidiomycete laccase gene families**.

**Strain**	**Laccase number**	**Protein length (aa)**	**Identification**	**Major laccase(s)**	**Expression analysis**	**Detection**	**References**
*Auricularia auricula-judae*	7	575–620	Transcriptome	Lcc3/5	Free-living and substrate mycelia and fruiting bodies	qRT-PCR	Fan et al., 2014
*Cerrena* sp. HYB07	8	516–542	Cloning	Lac2/7	Submerged fermentation	RT-PCR and LC-MS/MS	Yang et al., 2016a,b
*Coprinopsis cinerea*	17	516–567	Genome	Lcc1/5	Submerged fermentation	Zymograms and LC-MS/MS	Hoegger et al., 2004; Kilaru et al., 2006a; Rühl et al., 2013
*Flammulina velutipes*	11	502–607	Genome	Lac4	Mycelia and fruiting bodies	Transcriptome	Wang W. et al., 2015
*Laccaria bicolor*	9	504–540	Genome	LCC3/7	Mycelia, ectomycorrhizas and fruiting bodies	Custom whole-genome expression oligoarrays and qRT-PCR	Courty et al., 2009
*Lentinula edodes*	11	515–563	Genome	–	Mycelia, fruiting bodies	qRT-PCR	Sakamoto et al., 2015
*Pleurotus ostreatus*	11	516–541	Genome	Lacc2/10	Submerged fermentation, solid state fermentation, fruiting bodies	qRT-PCR	Castanera et al., 2012; Pezzella et al., 2013
*Trametes hirsuta* 072	5	519–523	cDNA library	LacA	Submerged fermentation	qRT-PCR	Vasina et al., 2015
*Trametes* sp. AH28-2	7	501–525^a^	Genome	LccA	Submerged fermentation	Native PAGE	Xiao et al., 2003, 2006; Zhang et al., 2015
*Volvariella volvacea*	11	508–562	Genome	Lcc3	Stipes	Digital gene expression (DGE) and qRT-PCR	Bao et al., 2013; Wu et al., 2013; Lu et al., 2015

a *The length of LacA-C*.

### Other laccase producers

Laccases also play diverse physiological roles in plants and bacteria, aside from metabolism of xenobiotics (Dwivedi et al., 2011; Singh et al., 2011; Chandra and Chowdhary, 2015; Forootanfar and Faramarzi, 2015; Wang J. et al., 2015). Plant laccase families are even larger than fungal laccase families. For example, there are at least 22 laccase genes in rice (*Oryza sativa*) (Huang et al., 2016). The model plant, *Arabidopsis thaliana*, has 17 members in its laccase gene family, which play several roles in plant growth and development, based on mutant characterization and expression profiling (Cai et al., 2006; Turlapati et al., 2011). Cotton contains 84, 44, and 46 laccase genes in cultivated allotetraploid *Gossypium hirsutum* and its two progenitor diploids *G. arboreum* and *G. raimondii* (Balasubramanian et al., 2016). In opposite to fungal laccases, plant laccases participate in lignin synthesis and therefore can be engineered for energy plant improvement (Wang J. et al., 2015). Plant laccases are also involved in pigmentation (Liang et al., 2006) or pigment breakdown (Fang F. et al., 2015), root elongation (Liang et al., 2006), and responses to external stresses (Cho et al., 2014; Kim et al., 2014).

Bacterial laccases were discovered relatively late compared to plant and fungal laccases (Ausec et al., 2011), but research on bacterial laccases have gained momentum over the past two decades. Bacterial laccases are implicated in various processes ranging from UV protection, pigmentation, metal oxidation, sporulation to xenobiotic degradation (Singh et al., 2011; Chandra and Chowdhary, 2015; Forootanfar and Faramarzi, 2015). Due to the widespread existence and versatility of bacteria, bacterial laccases have higher thermostability, alkaline pH optimum and halotolerance despite their low redox potentials and are valuable functional complements to fungal laccases in dye decolorization, pulp biobleaching, biofuel production as well as various other industrial and biotechnological fields (Santhanam et al., 2011; Singh et al., 2011; Chandra and Chowdhary, 2015; Martins et al., 2015).

Insect laccases are the least characterized of all known laccases. Insect laccases also play important roles in insect physiology such as cuticle sclerotization and melanization (Dittmer and Kanost, 2010; Jeon et al., 2012; Ni and Tokuda, 2013).

## Classical and molecular breeding for enhancing laccase production

### Classical breeding approaches

In addition to isolating natural, efficient laccase producers, classical and molecular breeding approaches are also used to increase laccase production. A successful example was provided by *N*-methyl-*N*-nitro-*N*-nitrosoguanidine and ultraviolet light treatments of *C. gallica* TCK. A mutated strain T906 was obtained, which showed three-fold higher laccase activity than the starting strain and a maximum laccase activity of 303 U/mL after 13 days (Xu et al., 2016). Mating of monokaryotic compatible *P. ostreatus* strains led to dikaryotic strains with higher laccase activity, and the best one produced 110 U/mL after induction for 8 days (Lettera et al., 2011; del Vecchio et al., 2012). The dikaryotic superiority in laccase activity is derived from non-additive increases in laccase transcription (Castanera et al., 2013). Furthermore, N^+^ ion implantation has recently been successfully applied to improve laccase production in *Paecilomyces* sp. WSH-L07 (Liu et al., 2010) and *Ceriporiopsis subvermispora* (Wang C. et al., 2012).

### Heterologous expression

Heterologous expression is invaluable in obtaining laccase proteins based only on metagenomic sequences (Beloqui et al., 2006; Fang et al., 2011, 2012). Heterologous expression is also important for isolating a laccase from other isozymes, especially when the enzyme is not abundantly expressed or silent. In addition to structural and biochemical characterization, heterologously expressed laccases can be engineered by rational design or directed evolution for enhanced expression, catalytic activity, stability, etc. The readers are referred to the recent reviews on laccase engineering (Rodgers et al., 2010; Alcalde, 2015; Mate and Alcalde, 2015; Pardo and Camarero, 2015). Enzyme resurrection, which is heterologous expression of ancestral enzymes reconstructed based on phylogenetic analysis and inference, is of particular interest (Alcalde, 2015). Ancestral enzymes are likely to have unique and extreme properties, such as greater stability and substrate promiscuity than extant ones, considering characteristics of ancient life (e.g., thermophilic) and generalist-specialist conversion of enzymes during the course of evolution (Risso et al., 2014). White-rot fungi and lignin degradation are dated back to the Permo-Carboniferous period (Floudas et al., 2012), therefore laccase resurrection brings an intriguing and promising toolset to laccase engineering and deserves more research efforts. The resurrected enzymes can then be subjected to further engineering by directed evolution (Alcalde, 2015).

The most common heterologous host is the methylotrophic yeast *Pichia pastoris* with its inducible *alcohol oxidase* (*AOX1*) promoter (Antošovǎ and Sychrová, 2016; Ergün and Çalık, 2016). Other hosts, such as prokaryotes (e.g., *E. coli* and *B. subtilis*), yeasts (e.g., *Saccharomyces cerevisiae* and *Yarrowia lipolytica*), filamentous fungi (e.g., *Trichoderma reesei* and *Aspergillus niger*), and plants (e.g., *Nicotiana tabacum* and *O. sativa*), are also used (Piscitelli et al., 2010; Kittl et al., 2012; Liebeton et al., 2014; Mate and Alcalde, 2015; Antošovǎ and Sychrová, 2016). In particular, heterologous laccases were constitutively expressed in basidiomycetes *Phanerochaete chrysosporium* (Coconi-Linares et al., 2015) and *C. cinerea* (Muraguchi et al., 2011). Although, filamentous fungi are efficient in protein secretion and have actually given rise to some of the highest recombinant laccase yields reported, genetic techniques are more readily available for yeasts (Piscitelli et al., 2010; Mate and Alcalde, 2015; Antošovǎ and Sychrová, 2016).

Expression systems like *P. pastoris* are used to produce enzymes at the industrial scale, but ligninolytic enzymes like laccases are notoriously difficult to express heterologously (Gu et al., 2014; Ergün and Çalık, 2016). Summaries of heterologously expressed laccases can be found in recent publications (Kittl et al., 2012; Mate and Alcalde, 2015; Antošovǎ and Sychrová, 2016; Ergün and Çalık, 2016). Occasionally, high recombinant laccase activity is obtained (Hong et al., 2002, 2007; Nishibori et al., 2013), but many recombinant laccases are expressed at levels below 10 U/mL, which can be even lower than that in the native strain (Yang et al., 2016b). An appropriate host is needed for heterologous expression of laccases, but “the best host” remains elusive (Piscitelli et al., 2010; Rivera-Hoyos et al., 2013). *Cryptococcus* sp. S-2 is a better yeast host than *P. pastoris* for expression of *T. versicolor* and *Gaeumannomyces graminis* laccase genes. The expression advantage is likely due to similar codon usage and GC content of *Cryptococcus* sp. S-2 with those of *T. versicolor* and *G. graminis* (Nishibori et al., 2013). Nonetheless, different codon preferences of the expression host and the gene source fails to explain the variability in production yields between laccase genes derived from the same organism (Piscitelli et al., 2010). Strategies employed to increase laccase production in heterologous systems include promoter and signal peptide selection, protein engineering, codon optimization, and optimization of cultivation medium composition and process. Since different and sometimes controversial results have been recorded, it is still difficult to predict the most promising combination of parameters to maximize heterologous laccase production (Piscitelli et al., 2010; Antošovǎ and Sychrová, 2016).

### Homologous expression

Due to low laccase yields in heterologous hosts, homologous expression in laccase-producing hosts might be of value for promoting laccase production. Homologous laccase expression has been attempted in *A. niger* (Ramos et al., 2011), *C. cinerea* (Kilaru et al., 2006b), *Gloeophyllum trabeum* (Arimoto et al., 2015), *P. cinnabarinus* (Alves et al., 2004), and *T. versicolor* (Kajita et al., 2004). For overexpression, the laccase gene is often driven by a strong promoter such as the constitutive glyceraldehyde-3-phosphate dehydrogenase gene (*gpd*) promoter (Alves et al., 2004; Kajita et al., 2004; Kilaru et al., 2006b; Arimoto et al., 2015) and maltose-induced glucoamylase gene (*glaA*) promoter (Ramos et al., 2011). In particular, when various basidiomycete promoters were compared, the *A. bisporus gpdII* promoter is more efficient in driving expression of the homolgous *Lcc1* gene in *C. cinerea* than the *C. cinerea tub1* (β-tubulin gene) promoter, *Lentinus edodes priA* (fruiting body gene) promoter or *S. commune Sc3* (hydrophobin gene) promoter (Kilaru et al., 2006b). The native laccase promoter was also used, and a high laccase production level of 1 g/L was achieved in the presence of 40 g/L ethanol after fermentation of transgenic *P. cinnabarinus* for 24 days (Alves et al., 2004).

## Regulation of laccase expression

Following successful screening of laccase-producing native hosts, laccase production is improved by fermentation technology development with respect to fermentation type, medium composition, and cultivation parameters (Elisashvili and Kachlishvili, 2009; Forootanfar and Faramarzi, 2015). Enhancing laccase yields is essential to lower production costs and promote industrial applications of the enzyme, which relies on understanding of laccase expression regulation. Numerous publications and reviews have been devoted to expression regulation of laccases (Piscitelli et al., 2011; Janusz et al., 2013).

Expression of laccase isozyme genes is differentially regulated throughout fermentation and in response to medium composition, such as metal ions, xenobiotics as well as nutrient types and levels. Laccase expression analysis has been performed on mRNA and protein levels, and the distinct responses of species, strains as well as genes no doubt paint a complex picture of laccase expression regulation. In accordance, various *cis*-acting responsive elements have been identified in laccase promoter regions, such as metal response element (MREs), ACE1 copper-responsive transcription factor binding sites (ACE1), xenobiotic response elements (XREs), antioxidant response elements (AREs), heat shock response elements (HSEs), CreA binding sites (CreA), and NIT2 binding sites (NIT2) (Piscitelli et al., 2011; Janusz et al., 2013). Nonetheless, function of most of the putative responsive elements is not experimentally validated, and how they interact with transcription factors remains elusive.

Copper ions are probably the most used inducer in laccase production, and the ACE1 binding site represents the most well-understood regulatory element in the laccase promoter region. Copper ions interact with the transcription factor ACE1 in *P. brumalis* (Nakade et al., 2013) or CUF1 in *C. neoformans* (Jiang et al., 2009) to increase laccase expression. In yeast, ACE1 and CUF1 have interchangeable *N*-terminal copper-fist DNA binding motifs despite opposite roles in maintaining copper homeostasis (Beaudoin et al., 2003). On the other hand, a copper-responsive laccase gene without an orthodox ACE1 binding site within its promoter might be regulated through a nonconventional copper-responsive element or a different mechanism (Yang et al., 2016a). Even when copper ions are not able to induce laccase production, they stabilize the copper-containing catalytic center of the enzyme (Solé et al., 2012), thus contributing to laccase activity. Besides copper, manganese and zinc are also commonly found to stimulate laccase synthesis (Lu and Ding, 2010; Solé et al., 2012; Yang et al., 2016a).

Literature describing laccase induction by xenobiotics, e.g., lignin breakdown products, dyestuffs and organic pollutants, has been accumulating. The effects of organic compounds on laccase production depend on the compound structure, fungal strain, and growth stage, laccase isozyme as well as the culture medium (Elisashvili et al., 2010; Giardina et al., 2010; Lu and Ding, 2010; Piscitelli et al., 2011; Solé et al., 2012; Janusz et al., 2013; Yang Y. et al., 2013). Combinational induction of laccase production by metal ions and organic compounds can be either synergistic (Yang Y. et al., 2013) or antagonistic (Lu and Ding, 2010).

Coculture of laccase-producing strains with other microbes is a natural way to induce laccase production, in the form of either yield increase or induction of new isozymes, and can be more effective than chemical induction. Microbial interactions with laccase inducing effects vary with the strain, but the structure of inducing metabolites and the inducing mechanism remain largely unknown (Zhang et al., 2006; Elisashvili and Kachlishvili, 2009; Flores et al., 2009; Wei et al., 2010; Pan et al., 2014; Li et al., 2016). One proposed mechanism for laccase overproduction in the coculture process is carbon source succession. Li et al. found that glycerol produced from glucose by the yeast *Candida* sp. is an efficient carbon source for *G. lucidum* upon glucose deprivation and crucial for laccase overproduction by prolonging laccase secretion time (Li et al., 2011). Phenolics and lysing enzymes produced by opposing microbes have also been suggested to have laccase-inducing ability (Zhang et al., 2006; Wei et al., 2010).

Nutrient types and concentrations have been extensively studied in the context of fungal growth and enzyme secretion. Since basidiomycetes display a wide diversity in their responses, no generalization can be made on the best carbon and nitrogen sources or their optimal concentrations (Elisashvili and Kachlishvili, 2009; Piscitelli et al., 2011; Janusz et al., 2013). Lignocellulosic wastes containing carbohydrates and inducers are often added resulting in benefits such as lower production costs, waste reuse, and laccase production enhancement (Elisashvili and Kachlishvili, 2009; Postemsky et al., 2017).

In many fungi, laccase is produced by secondary metabolism, that is, laccase synthesis is activated by carbon or nitrogen depletion. This no doubt necessitates a long production cycle and encumbers industrial production of laccase. Therefore, a promising commercial laccase producer should produce laccase with high yields and a short fermentation cycle. A recently reported *Cerrena* sp. HYB07 is an example of such laccase producers (Yang et al., 2016a). Its laccase production is not inhibited by high nutrient levels, which allows biomass accumulation and a quick peak of laccase activity (Table 1). Furthermore, the laccase yield of HYB07 is mostly attributed to the predominantly expressed *Lac7*. The strength of the *Lac7* promoter requires only copper ions and high nutrient concentrations, but not aromatic inducers, making it interesting for recombinant expression of other laccase genes.

Other factors on laccase expression are studied to a lesser extent. Laccase expression could also be regulated by oxidative stress (Yang et al., 2012; Si and Cui, 2013; Fernandez-Alejandre et al., 2016), heat shock (Wang F. et al., 2012), cAMP (Crowe and Olsson, 2001), and calmodulin (Suetomi et al., 2015).

Apparently, crosstalk exists between the internal factors (e.g., fungal strain, growth stage, laccase promoter, etc.) and external factors (metal ions, organic compounds, nutrient sources, and ratios, etc.) influencing laccase synthesis. However, until now, the bulk of the work has only attempted to decipher regulation of laccase expression by an isolated single factor or a few factors, which is undoubtedly a simplification of the complex network controlling laccase expression. The mechanism underlying the regulatory network of laccase expression awaits elucidation.

## Laccase mediators

The efficiency of substrate oxidation by a laccase depends on the difference between the redox potentials of the substrate and the T1 Cu. Due to the lower redox potentials of laccases (≤0.8 V) compared to ligninolytic peroxidases (>1 V) (Wong, 2009; Rivera-Hoyos et al., 2013; Pollegioni et al., 2015; Sitarz et al., 2016), laccases are originally thought to be able to oxidize only the phenolic lignin moiety, with the majority of lignin being non-phenolic and with higher redox potentials. Low-molecular-weight redox mediators are used to expand the laccase substrate range or increase the reaction rate, especially for substrates with higher redox potentials or too large to fit in the enzyme's active site. Commonly used laccase mediators include synthetic mediators such as 2,2′-azino-bis (3-ethylbenzothiazoline-6-sulfonate) (ABTS) and 1-hydroxybenzotriazole (HBT) as well as natural phenolic mediators such as syringaldehyde (SA) and acetosyringone (AS). Despite the proven efficiency of artificial mediators, natural mediators (believed to be true mediators of fungal laccases in nature) are considered to be alternatives to the artificial ones because they are more economically feasible and environmentally friendly (Cañas and Camarero, 2010). Laccase oxidation of the substrate may proceed differently with a mediator. However, it is not always the case. Malachite green degradation products in the presence and absence of ABTS have been shown to be identical or different, depending on the enzyme (Chhabra et al., 2009; Yang et al., 2015).

Different types of mediators have different catalytic mechanisms. ABTS-mediated substrate oxidation proceeds via an electron transfer route. ABTS is first oxidized to its radical cation (ABTS^·+^) and then to the di-cation (ABTS^2+^) with redox potentials of 472 and 885 mV, respectively. Unlike ABTS, an N-OH type mediator (such as HBT and violuric acid) forms the N-oxy radical upon laccase oxidation and subsequent deprotonation; the radical in turn abstracts the benzylic hydrogen atom from the substrate. Similarly, phenolic mediators also follow a radical hydrogen abstraction mechanism, but with the intermediate being a phenoxy radical (Hu et al., 2009; Wong, 2009). The effect of a mediator on laccase oxidation varies with the laccase and substrate and depends on the radicals formed, recyclability of the mediator and stability of the laccase in the presence of the mediator (Morozova et al., 2007; Wong, 2009; Cañas and Camarero, 2010; Pogni et al., 2015). Regardless of the reaction mechanism, mediators incur additional costs, and can cause toxicity (Weng et al., 2013; Becker et al., 2016) and laccase inactivation (Kurniawati and Nicell, 2007; Fillat et al., 2012; Ashe et al., 2016). Although, laccases without the requirement for facilitating mediators, the laccase/mediator system is regarded as a feasible industrial solution, ideal mediators that are cheap, green, effective, stable, recyclable, not toxic, or enzyme-inactivating should be ascertained (Morozova et al., 2007; Kües, 2015).

## Laccase immobilization

Laccases are immobilized for recycling, operational stability, and resistance to application conditions. Immobilization techniques include entrapment, adsorption, covalent binding, self-immobilization as well as combinations of the aforementioned techniques. Activity recovery varies based on the enzyme, the immobilization method of choice, and preparation parameters. Compared with their free counterparts, immobilized laccases are more tolerant to high temperatures and storage and can be reused multiple times (Fernández-Fernández et al., 2013; Asgher et al., 2014), they are also more resistant to inhibitors such as NaCl (Yang et al., 2016c). Immobilization sometimes improves the catalytic activity of laccases (Arsenault et al., 2011; Sinirlioglu et al., 2013; Kumar et al., 2014) despite the common concern of reduced enzyme flexibility, steric hindrance and diffusion limitations (Sheldon, 2011; Talekar et al., 2013). Readers can refer to reviews on preparation and applications of immobilized laccases (Ba et al., 2013; Fernández-Fernández et al., 2013; Asgher et al., 2014).

## Laccase applications in biodegradation of PPCPs

The value of fungi as well as fungal enzymes in pollution control and environment management has been recognized. Examples of environmentally important enzymes comprise hydrolases, laccases, lyases, peroxidases, tyrosinases, and P450 cytochrome monooxidases (Demarche et al., 2012; Yang et al., 2013a; Rao et al., 2014; Kües, 2015; Yadav and Yadav, 2015; Martinkova et al., 2016). The ability of laccases to effectively degrade and detoxify a variety of persistent organic pollutants (POPs) has received considerable attention in the field of bioremediation (Majeau et al., 2010; Strong and Claus, 2011; Gasser et al., 2014; Viswanath et al., 2014; Catherine et al., 2016), and laccases can also be used in enzymatic biosensors for environmental pollution monitoring (Rao et al., 2014). A summary of environmental contaminants as laccase substrates from published research in the year 2016 is provided in Table 3. The contaminants investigated include dyestuffs (Singh R. L. et al., 2015; Sen et al., 2016), polycyclic aromatic hydrocarbons (PAHs) (Librando and Pappalardo, 2013), endocrine disrupters (Cabana et al., 2007; Husain and Qayyum, 2012; Gasser et al., 2014), and pesticides (Maqbool et al., 2016).

**Table 3:** **Laccase applications in biodegradation and bioremediation in 2016**.

**Compound**	**Laccase**	**Enzyme form**	**Mediator**	**References**
**PHENOLS**				
Chlorophenols, cresols, nitrophenols	*Trametes sanguineus* laccase expressed in *Trichoderma atroviride*	In culture	–	Balcazar-Lopez et al., 2016
Technical nonylphenol	*Phoma* sp. UHH 5-1-03	Free	SA	Hofmann and Schlosser, 2016
Oxybenzone, pentachlorophenol	*P. ostreatus*	Free	ABTS, HBT, HPI, TEMPO, SA, VA, VAN	Ashe et al., 2016
4-tert-butylphenol, 4-tert-octylphenol	*Myceliophthora thermophila* laccase expressed in *Aspergillus oryzae* (Novozyme)	Enzymatic membrane reactor	SA	Nguyen et al., 2016b
2,4-dichlorophenol	*Pycnoporus sanguineus* CS43	Free	–	Rodríguez-Delgado et al., 2016
**DYESTUFFS**				
Bromophenol Blue, Congo Red, Coomassie Blue, Tripan Blue	*T. sanguineus* laccase expressed in *T. atroviride*	Free	–	Balcazar-Lopez et al., 2016
Acid Black 172, Congo Red, Crystal Violet, Direct Fast Blue FBL, Indigo Blue, Naphthol Green B, Methylene Blue, Neutral Red, Reactive Brilliant Blue X-BR, Remazol Brilliant Blue Reactive (RBBR)	*T. pubescens*	Chitosan beads	–	Zheng et al., 2016
Acid Orange 67, Basic Red 18, Basic Yellow 28, Direct Black 166, Direct Yellow 107, Disperse Yellow 79	*Paraconiothyrium variabile*	Free	HBT	Forootanfar et al., 2016
Brilliant Blue G, Brilliant Blue R, Bromophenol Blue, Coomassie Blue R250, Crystal Violet, Malachite Green, Methylene Blue, Xylene Cyanol, RBBR	*P. sanguineus*	Free	VA	Iracheta-Cárdenas et al., 2016
RBBR	*T. versicolor* (Sigma-Aldrich)	Core-shell magnetic copper alginate beads	HBT	Le et al., 2016
RBBR	*Cerrena* sp. HYB07	Cross-linked enzyme aggregates	–	Yang et al., 2016c
Coomassie Blue R250	*Cerrena* sp. HYB07	Free	ABTS, AS, HBT, SA, SYA	Yang et al., 2016d,e
**ENDOCRINE DISRUPTERS**				
Bisphenol A (BPA)	*Coriolopsis gallica, Bjerkandera adusta, T. versicolor*	Free	HBT	Daâssi et al., 2016
BPA	*T. sanguineus* laccase expressed in *T. atroviride*	In culture	–	Balcazar-Lopez et al., 2016
BPA	*T. versicolor* laccase expressed in *S. cerevisiae*	Surface display	ABTS	Chen et al., 2016
BPA	*T. versicolor* (Sigma-Aldrich)	Cross-linked carbon nanotubes-based biocatalytic membranes	–	Ji et al., 2016a
BPA	*M. thermophila* laccase expressed in *A. oryzae* (Novozyme)	On granular activated carbon, continuous flow packed-bed reactor	–	Nguyen et al., 2016a
BPA, 17α-ethinylestradiol	*T. versicolor*	Polyamide 6/chitosan nanofibers	–	Maryskova et al., 2016
BPA, 17α-ethinylestradiol	*Phoma* sp. UHH 5-1-03	Free	SA	Hofmann and Schlosser, 2016
BPA, 17α-ethinylestradiol, 17α-estradiol, 17α-estradiol 17–acetate, estriol, estrone	*M. thermophila* laccase expressed in *A. oryzae* (Novozyme)	Enzymatic membrane reactor	SA	Nguyen et al., 2016b
**POLYCYCLIC AROMATIC HYDROCARBONS (PAHs)**				
All 15 US EPA priority PAHs	*B. subtilis* CotA expressed in *E. coli*	Free	ABTS	Zeng et al., 2016
Naphthalene, phenanthrene	*T. versicolor* (Sigma-Aldrich)	Nonionic surfactant-modified clay	–	Chang et al., 2016
Benzo[a]pyrene, phenanthrene	*T. sanguineus* laccase expressed in *T. atroviride*	Free	–	Balcazar-Lopez et al., 2016
**PESTICIDES**				
Atrazine	*P. ostreatus*	Free	ABTS, HBT, HPI, TEMPO, SA, VA, VAN	Ashe et al., 2016
Chlorpyrifos	Bacterial WlacD expressed in *Pseudomonas putida*	Surface display	–	Liu et al., 2016
Atrazine, chlorothalonil, chlorpyrifos, isoproturon, pyrimethanil	*T. versicolor*	Free	ABTS, AS, guaiacol, HBT, SA, VA, VAN	Jin et al., 2016
Atrazine, isoproturon	*O. sativa* laccases expressed in *P. pastoris*	In culture	–	Huang et al., 2016
Ametryn, atrazine, clofibric acid, fenoprop, pentachlorophenol, propoxur	*M. thermophila* laccase expressed in *A. oryzae* (Novozyme)	Enzymatic membrane reactor	SA	Nguyen et al., 2016b
**MYCOTOXINS**				
Aflatoxin B1 and M1	*P. pulmonarius*	Free	ABTS, AS, SA	Loi et al., 2016

*All tested mediators are listed. ABTS, 2,2′-azino-bis (3-ethylbenzothiazoline-6-sulfonate); AS, acetosyringone; HBT, 1-hydroxybenzotriazole; HPI, N-hydroxyphthalimide; TEMPO, 2,2,6,6-tetramethylpiperidinyloxyl; SA, syringaldehyde; SYA, syringic acid; VA, violuric acid; VAN, vanillin*.

Pharmaceuticals and personal care products (PPCPs) are becoming ubiquitous in the environment and are recognized as emerging trace organic contaminants (Onesios et al., 2009; Oulton et al., 2010; Wang and Wang, 2016). Laccases can be employed for their removal (Gasser et al., 2014). Laccases have been used in PPCPs as an ingredient; many products generated by laccases have antimicrobial, anticancer, antioxidant, detoxifying, or other activities (Senthivelan et al., 2016; Upadhyay et al., 2016). Specifically, laccases can be used to synthesize novel antibiotics (Mikolasch et al., 2012, 2016; Pezzella et al., 2015), and laccase-based antimicrobial formulations are considered a safe and green alternative to chemical decontamination (Grover et al., 2013). Nonetheless, the focus of this review lies in the degradation and detoxification of PPCP contaminants with laccases.

### Degradation of antibiotics

Antibiotics constitute one of the most used classes of drugs in the world; they are used in human and veterinary medicine as well as livestock farming. Antibiotics that are not metabolized enter the environment (Larsson, 2014). Conventional water treatment processes cannot effectively remove antibiotics (Oulton et al., 2010), while more efficient advanced treatment methods have disadvantages such as high costs and secondary pollution (Chen et al., 2016). Antibiotics pose health risks by selecting for antibiotic-resistance bacteria (ARB). Antibiotics, ARB, and antibiotic-resistant genes have been detected in soil, sediments, and water bodies including wastewater drinking water and marine water (Thiele-Bruhn, 2003; Kümmerer, 2009; Guo et al., 2014; Larsson, 2014). There has been a fast growth in the literature describing laccase utilization in antibiotic removal, especially within the past 2 years, but this topic has not been properly reviewed.

Target antibiotics under investigation include penicillins, tetracyclines, sulfonamides, quinolones and trimethoprim, and sulfamethoxazole and tetracycline are two most studied (Table 4). The removal time ranges from minutes to hours, depending on the laccase, antibiotic and treatment parameters. Mediators such as HBT, ABTS, and SA are often used to enable or accelerate antibiotic conversion by laccases. In fact, significant antibiotic removal within 1 h usually requires involvement of an appropriate mediator (Suda et al., 2012; Weng et al., 2012, 2013; Shi et al., 2014; Ding et al., 2016). Manganese peroxidase was more efficient in tetracycline conversion than laccase, but the addition of HBT can promote laccase catalysis to a rate higher than that of manganese peroxidase (Wen et al., 2010; Suda et al., 2012) although still slower than that of lignin peroxidase (95% degradation efficiency in 5 min; Wen et al., 2009). Interestingly, mediators, i.e., ABTS, SA, and AS, are consumed without observed catalytic activity during degradation of sulfamethoxazole (Margot et al., 2015).

**Table 4:** **Laccase treatment of antibiotics**.

**Compound**	**Laccase**	**Enzyme form**	**Reaction parameters**	**Efficiency**	**Toxicity after treatment**	**References**
**PENICILLINS**						
Amoxicillin, ampicillin, cloxacillin, penicillin G, penicillin V, oxacillin	*T. versicolor* (Sigma-Aldrich)	Enzymatic membrane reactor	10 μg/L antibiotics, 1 mM SA, starting pH 6, 25°C, 0.07 m/s flow, tangential configuration	54–100% after 24 h	Increased (*B. subtilis* and *V. fischeri)*	Becker et al., 2016
**SULFONAMIDES**						
Sulfapyridine, sulfathiazole	*T. versicolor* (Sigma-Aldrich)	Free	16–20 mg/L antibiotic, 50–55 U/L laccase, 0.8 mM VA, pH 4.5, 25°C, 135 rpm	100% after 8 h	NR	Rodriguez-Rodriguez et al., 2012
Sulfadimethoxine, sulfamonomethoxine	*Perenniporia* strain TFRI 707	Free	50 mg/L antibiotic, 6 U/mL, 1 mM ABTS or VA, pH 4.1, 30°C, 8% glycerol	*t*_(1/2)_ (min): 1.8–4.1	NR	Weng et al., 2012
Sulfadimethoxine, sulfamonomethoxine	*Perenniporia* strain TFRI 707	Free	50 mg/L antibiotic, 6 U/mL laccase, 8% glycerol; 1 mM ABTS, pH 4, 50–60°C; 1 mM VA, pH 4, 40–60°C; 2 mM SA, pH 6, 50°C	100% after 30 min with ABTS; 100% after 15 min with VA; >95% after 60 min with SA	Reduced (*V. fischeri*) with VA and HBA; increased with ABTS and SA	Weng et al., 2013
Sulfamethoxazole	*T. versicolor*	Free	1,100 μg/L antibiotic, 1 mM HBT, 25°C, 70 rpm	41% after 22 h	NR	Yang et al., 2013b
Sulfamethoxazole	*M. thermophila* laccase expressed in *A. oryzae* (Novozyme)	Enzymatic membrane reactor	830 μg/L d antibiotic, 70–100 μM/min laccase, 5 μM SA, 3 g/L granular activated carbon	65%	Increased (ToxScreen3 assay with *Photobacterium leiognathi*), which can be reduced by granular activated carbon addition	Nguyen et al., 2014b
Sulfadiazine, sulfamethazine, sulfamethoxazole	*Echinodontium taxodii*	Oriented immobilization on Fe_3_O_4_ nanoparticles	50 mg/L antibiotic, 0.2 U/mL laccase, 1 mM AS, SA or SYA, pH 5	>95% after 30 min	Reduced (*E. coli* and *S. aureus*)	Shi et al., 2014
Sulfamethoxazole	*T. versicolor* (Sigma-Aldrich)	Free	73–93 μM antibiotic, mediator/laccase ratio: 1.1 (ABTS), 1.7 (SA) or 2.4 (AS), pH 6, 25°C, static	100%	Reduced (algae *Pseudokirchneriella subcapitata*)	Margot et al., 2015
Sulfamethoxazole, sulfathiazole	*T. versicolor*	On porous silica beads	50 mg/L antibiotic, 1 U/mL laccase, 1 mM HBT, pH 5, 40°C, 50 rpm	76–85% after 1 h	Reduced (*E. coli, P. aeruginosa, H. influenza, S. enterica, S. aureus, S. pneumoniae*)	Rahmani et al., 2015
Sulfadimethoxine	*T. versicolor*	Free	Per gram soil: 2 μg antibiotic, 10 U laccase, 8 μmol ABTS or HBT, 1 mg peat; room temperature	>90% after 72 h	NR	Singh R. et al., 2015
Sulfadiazine, sulfamethoxazole,	*P. sanguineus*	In culture	25 mg/L antibiotics, 600 U/L laccase, 0.274 g/L ABTS, 28°C	84% after 6 h	Reduced (*E. coli, B. subtilis, B. licheniformis*)	Li et al., 2016
Sulfamethoxazole	*T. versicolor* Lac3 expressed in *S. cerevisiae*	Surface display	30 μM antibiotic, 0.25 U/mL laccase, pH 5, 37°C, 250 rpm	44% after 30 h	NR	Chen et al., 2016
Sulfamethoxazole	*Phoma* sp. UHH 5-1-03	Free	250 μM antibiotic, 3 U/mL laccase, 500 μM SA, pH 5, 22°C, 120 rpm	87% after 22 h	NR	Hofmann and Schlosser, 2016
Sulfabenzamide, sulfadiazine, sulfadimethoxine, sulfamerazine, sulfamethizole, sulfamethoxazole, sulfamethoxypyridazine, sulfanitran, sulfapyridine, sulfisomidine, sulfisoxazole, sulfathiazole,	*T. versicolor* (Sigma-Aldrich)	Enzymatic membrane reactor	10 μg/L antibiotics, 10 or 1,000 μM SA, starting pH 6, 25°C, 0.07 m/s flow, tangential configuration	43–97% after 24 h with 10 μM SA; 50–100% after 24 h with 1,000 μM SA	Increased (*B. subtilis* and *V. fischeri)*	Becker et al., 2016
Sulfamethoxazole	*M. thermophila* laccase expressed in *A. oryzae* (Novozyme)	On granular activated carbon, continuous flow packed-bed reactor	0.5 mg/L antibiotic, 0.4 g/mL laccase, 28°C, 8.5 BV/h (BV: 17 mL)	100% for 4,000 BV, 70% after 12,000 BV (60 d)	NR	Nguyen et al., 2016a
Sulfadiazine, sulfamethazine, sulfamethoxazole, sulfapyridine, sulfathiazole	*T. versicolor* (Sigma-Aldrich)	Free	10 mg/L antibiotics, 0.5 mg/mL laccase, 0.5 mM SA, pH 6, 25°C, 200 rpm	73–80% after 15 min; 97–99% after 180 min	NR	Ding et al., 2016
**TETRACYCLINES**						
Chlortetracycline, doxycycline, oxytetracycline, tetracycline	*T. versicolor*	Free	100 μM antibiotic, 10 nkat/mL laccase, 0.2 mM HBT, pH 4.5, 30°C, 150 rpm	Chlortetracycline and doxycycline: 100% after 15 min; tetracycline and oxytetracycline: 100% after 1 h	Reduced (*E. coli* and *Bacillus subtilis*)	Suda et al., 2012
Tetracycline	*T. versicolor* (Sigma-Aldrich)	Enzymatic membrane reactor	20 mg/L antibiotic, 0.002 g/L laccase, pH 6, 25°C, batch	0.34 mg/h for 10 d	NR	de Cazes et al., 2014
Tetracycline	*T. versicolor* (Sigma-Aldrich)	Enzymatic membrane reactor	20 mg/L antibiotic, 10 g/L laccase, 1.4 μm pore size, 25 cm length, tangential (10 L/h), 25°C, 8 L/h/m^2^ permeation	>200 mg/h/m^2^ for 24 h	NR	de Cazes et al., 2015
Tetracycline	*T. versicolor* (Sigma-Aldrich)	Free	100 μg/mL antibiotic, 17.5 μg/mL laccase, pH 7, 20°C	78% after 18 h	Reduced (*B*. *subtilis*)	Llorca et al., 2015
Chlortetracycline, doxycycline, oxytetracycline, tetracycline	*T. versicolor* (Sigma-Aldrich)	Enzymatic membrane reactor	10 μg/L antibiotics, 10 μM SA, starting pH 6, 25°C, 0.07 m/s flow, tangential configuration	85–98% after 24 h	Increased (*B. subtilis* and *V. fischeri*)	Becker et al., 2016
Chlortetracycline, doxycycline, oxytetracycline, tetracycline	*T. versicolor* (Sigma-Aldrich)	Free	10 mg/L antibiotics, 0.5 mg/mL laccase, 0.5 mM SA, pH 6, 25°C, 200 rpm	61–100% after 15 min; 95–100% after 180 min	NR	Ding et al., 2016
Oxytetracycline, tetracycline	*Cerrena* sp. HYB07	Magnetic cross-linked enzyme aggregates	100 μg/mL antibiotic, 40 U/mL laccase, pH 6, 25°C	80% after 12 h	Reduced (*E. coli* and *B. licheniformis*)	Yang et al., 2017
**QUINOLONES**						
Flumequine	*T. versicolor* (Sigma-Aldrich)	Free	90 mg/L antibiotic, 6 U/mL laccase, 1.35 mM ABTS, pH 4, 39°C, 150 rpm	98% after 2 h	NR	Ashrafi et al., 2015
Ciprofloxacin	*A. oryzae*	Free	10 mg/L antibiotic, 0.02% (w/v) laccase, pH 6, 60°C, 200 rpm ultrasound (75 W, 22 kHz, 50% duty cycle),	51% after 5 h	NR	Sutar and Rathod, 2015
Cinoxacin, ciprofloxacin, danofloxacin, difloxacin, enoxacin, enrofloxacin, flumequine, marbofloxacin, nalidixic acid, norfloxacin, ofloxacin, orbifloxacin, oxolinic acid, pipemidic acid	*T. versicolor* (Sigma-Aldrich)	Enzymatic membrane reactor	10 μg/L antibiotics, 10 or 1,000 μM SA, starting pH 6, 25°C, 0.07 m/s flow, tangential configuration	0–84% after 24 h with 10 μM SA; 15–93% after 24 h with 1,000 μM SA	Increased (*B. subtilis* and *V. fischeri)*	Becker et al., 2016
Ciprofloxacin, enoxacin, enrofloxacin, norfloxacin, ofloxacin	*T. versicolor* (Sigma-Aldrich)	Free	Laccase/SA (10 mg/L antibiotics, 0.5 mg/mL laccase, 0.5 mM SA, pH 6, 25°C, 200 rpm) coupled with soil (0.05 g/mL) adsorption	91–99% after 15 min; 96–100% after 180 min; enoxacin: 74% after 15 min with only laccase and SA	NR	Ding et al., 2016
Ciprofloxacin, norfloxacin	*Streptomyces ipomoeae* SilA expressed in *E. coli*	Free	50 mg/L antibiotic, 0.4 U/mL laccase, 0.5 mM AS, pH 8, 35°C	>70% after 4 h; >90% after 24 h	Reduced (*Pseudokirchneriella subcapitata*)	Blánquez et al., 2016
**DIHYDROFOLATE REDUCTASE INHIBITOR**						
Trimethoprim	*T. versicolor* (Sigma-Aldrich)	Enzymatic membrane reactor	10 μg/L antibiotic, 1 mM SA, starting pH 6, 25°C, 0.07 m/s flow, tangential configuration	66.8% after 24 h	Increased (*B. subtilis* and *V. fischeri)*	Becker et al., 2016
Trimethoprim	*T. versicolor* (Fluka)	Magnetic cross-linked enzyme aggregates	100 μg/L antibiotic, 1 U/mL laccase, 0.1 mM ABTS, pH 7, 20°C, 125 rpm	47% after 6 h; 60% after 12 h	NR	Kumar and Cabana, 2016
**NITROIMIDAZOLE**						
Metronidazole	*T. versicolor* (Sigma-Aldrich)	Enzymatic membrane reactor	10 μg/L antibiotic, 10 μM SA, starting pH 6, 25°C, 0.07 m/s flow, tangential configuration	25.9% after 24 h	Increased (*B. subtilis* and *V. fischeri)*	Becker et al., 2016

*Only the best reaction parameters are shown. ABTS, 2,2′-azino-bis (3-ethylbenzothiazoline-6-sulfonate); AS, acetosyringone; BV, bed volume; HBA, 4-hydroxybenzyl alcohol; HBT, 1-hydroxybenzotriazole; NR, not reported; SA, syringaldehyde; SYA, syringic acid; VA, violuric acid*.

Sulfonamides and tetracyclines are more easily attacked by laccase compared with quinolones (Becker et al., 2016; Ding et al., 2016). This is presumably due to the strong electron donating aromatic amine group in sulfonamides and the phenol group in tetracyclines, which are not found in quinolones (Ding et al., 2016). However, identified tetracycline transformation intermediates suggest that the phenol group is not the primary target for laccase oxidation, and that oxygen addition, demethylation, water elimination reactions occur during laccase treatment (Llorca et al., 2015; Yang et al., 2017). For sulfonamides, increasing electronegativity of the substituents is accompanied by decreased degradation (Yang C. W. et al., 2016). Two sulfonamides, namely sulfapyridine and sulfathiazole, are desulfonated by laccase (Rodriguez-Rodriguez et al., 2012). Covalent cross-coupling of sulfonamides is observed with laccase and mediator SA or AS (Shi et al., 2014; Margot et al., 2015), but not ABTS (Margot et al., 2015). Trimethoprim has 2 amine groups and 3 methoxy groups and is usually administered in combination with sulfamethoxazole. Little (Touahar et al., 2014; Arca-Ramos et al., 2016) to over 60% (Kumar and Cabana, 2016) degradation of this antibiotic without a mediator have been reported. Furthermore, SA at 1,000 μM, but not 10 μM, increases trimethoprim removal from 27 to 67%; nearly complete elimination of sulfamethoxazole is achieved under the same conditions (Becker et al., 2016). Some antibiotics (e.g., penicillins) are unstable in aqueous solutions, and attention should be paid to sample preservation and quantification (Llorca et al., 2014; Becker et al., 2016).

Laccase from *T. versicolor*, especially the product sold by Sigma-Aldrich, is most frequently used in biodegradation studies of antibiotics as well as other trace organic contaminants. Other laccases include laccases from basidiomycetes *Cerrena* sp. HYB07, *Echinodontium taxodii, Perenniporia* strain TFRI 707, and *P. sanguineus*, from ascomycetes *Phoma* sp. and *Myceliophthora thermophila* (recombinantly expressed in *Aspergillus oryzae*) and from actinobacteria *Streptomyces ipomoeae* (expressed in *E. coli*; Table 4). Laccases immobilized by different methods have been used for antibiotic degradation, including enzymatic membrane reactors (Nguyen et al., 2014b; Becker et al., 2016), granular activated carbon (Nguyen et al., 2016a), silica beads (Rahmani et al., 2015), oriented immobilization (Shi et al., 2014), magnetic cross-linked enzyme aggregates (Kumar and Cabana, 2016; Yang et al., 2017), and cell surface display (Chen et al., 2016). In particular, enzymatic membrane reactors (gelatin-ceramic membranes grafted with commercial *T. versicolor* laccase) in tetracycline degradation have been evaluated in depth with respect to membrane preparation, efficiency, kinetics, and economics (de Cazes et al., 2014, 2015; Abejón et al., 2015a,b). Mathematical cost estimation indicates that the enzymatic process is still economically uncompetitive. Improvements should be made in terms of enzyme kinetics, reactor effective lifetime and regeneration costs (Abejón et al., 2015a). For example, a pore diameter of 1.4 μm, in contrast to 0.2 μm, increases enzyme loading of the membrane reactor, avoids extensive membrane area, and facilitates tetracycline degradation (de Cazes et al., 2015).

Occasionally, laccases do not participate in antibiotic removal by white-rot fungi; for instance, laccase was not responsible for oxytetracycline degradation by *P. ostreatus* or *T. versicolor* (Migliore et al., 2012; Mir-Tutusaus et al., 2014) or sulfamethoxazole degradation by aquatic ascomycete *Phoma* sp. UHH 5-1-03 (Hofmann and Schlosser, 2016). In these cases, other enzymes, such as cytochrome P450, may be resorted to for biodegradation. It should still be pointed out that even when extracellular laccase is not able to directly oxidize sulfamethoxazole, when a mediator is added, significant removal is achieved (Yang et al., 2013b; Hofmann and Schlosser, 2016).

Laccases are also applied in combination with other processes in antibiotic treatment, such as ultrasound (Sutar and Rathod, 2015) and soil adsorption (Ding et al., 2016). The involvement of other processes facilitates degradation of antibiotics, e.g., quinolone antibiotics, which are recalcitrant to laccase oxidation. Laccase can also improve efficiency and stability of antibiotic removal by other organisms. When sulfamethoxazole is the transformed by non-laccase-producing bacteria *Alcaligenes faecalis*, the efficiency drops when some metabolites such as N4-acetyl-sulfamethoxazole are transformed back to the parent compound. The removal efficiency does not decrease when the coculture of *A. faecalis* with laccase-producing *P. sanguineus* is used or when cell-free laccase was added to *A. faecalis* culture (Li et al., 2016).

Toxicity of antibiotics after laccase treatment is commonly assessed via growth inhibition assay or bioluminescence inhibition test (Table 4). Antibiotic degradation by laccase mostly leads to reduced toxicity. A good example comes from the comparison of the sulfamethoxazole transformation products and their toxicity by *A. faecalis* with or without exogenous laccase. N-hydroxy sulfamethoxazole (HO-SMX), a toxic and recalcitrant intermediate of sulfamethoxazole, is formed upon *A. faecalis* treatment. Additional laccase, on the other hand, eliminates HO-SMX along with the toxicity (Li et al., 2016). However, sometimes laccase/mediator-catalyzed antibiotic transformation results in even higher toxicity, and this seems to frequently associate with the mediator SA (Weng et al., 2013; Nguyen et al., 2014b; Becker et al., 2016). It is postulated that the enhanced toxicity can be derived from oxidation of aromatic structures, especially phenols, to quinonoids (Becker et al., 2016).

The majority of studies on antibiotic degradation were carried out in aqueous environments, but there have been a few studies on remediation of soil (Singh R. et al., 2015), river sediment (Chang and Ren, 2015), and sludge (Yang C. W. et al., 2016). Laccase-containing extract from spent mushroom compost of *Pleurotus eryngii* and extract-containing microcapsules enhanced degradation of three tetracyclines in river sediment (Chang and Ren, 2015) as well as degradation of three sulfonamides in sewage sludge (Yang C. W. et al., 2016). Sulfonamide antibiotics can form stable covalent bonds with humic constituents, and laccase can catalyze unreactive hydroquinone moieties in humic acid to reactive, electrophilic quionone moieties which in turn react with the antibiotic. This will affect the fate, bioactivity, and extractability of sulfonamides in soils (Gulkowska et al., 2012, 2013; Schwarz et al., 2015).

### Degradation of other PPCPs

Besides antibiotics, many other PPCPs are actively evaluated as laccase substrates, including anticonvulsants (e.g., carbamazepine and benzodiazepines) (Ostadhadi-Dehkordi et al., 2012), fungicides (e.g., ketoconazole) (Yousefi-Ahmadipour et al., 2016), anti-inflammatory drugs (e.g., acetaminophen, aspirin, diclofenac, and ketoprofen) (Marco-Urrea et al., 2010a; Ba et al., 2014a; Domaradzka et al., 2015; Singh et al., 2016), antidepressants (e.g., imipramine) (Tahmasbi et al., 2016), lipid regulators (e.g., clofibric acid) (Ji et al., 2016a), biocides (triclosan and chlorophene) (Shi et al., 2016), insect repellents (e.g., *N*,*N*-diethyl-m-toluamide) (Tran et al., 2013), and sunscreen agents (e.g., oxybenzone) (Garcia et al., 2011).

Among these PPCPs, diclofenac, carbamazepine, and triclosan are the most investigated (Table 5); triclosan is phenolic and the other two are non-phenolic. Carbamazepine is the most recalcitrant to oxidation by laccase (Yang et al., 2013b; Nguyen et al., 2014a,b; Touahar et al., 2014; Hofmann and Schlosser, 2016; Kumar and Cabana, 2016) or peroxidases (Zhang and Geißen, 2010; Eibes et al., 2011). Carbamazepine contains a strong electron-attracting amide group, which may account for its recalcitrance (Yang et al., 2013b). In contrast, triclosan has a strong electron donating hydroxyl group despite simultaneous presence of electron withdrawing chlorinated groups, which makes it susceptible to laccase oxidation (Garcia-Morales et al., 2015). Chlorine atoms are also found in diclofenac along with aromatic amine (Nguyen et al., 2014c, 2015), but it is more prone to laccase oxidation than carbamazepine.

**Table 5:** **Laccase transformation of diclofenac, carbamazepine, and triclosan since 2010**.

**Compound**	**Laccase**	**Enzyme form**	**Mediator**	**Efficiency**	**References**
Diclofenac (anti-inflammatory)	*T. versicolor, T. versicolor* (Sigma-Aldrich)	Free	–	100% after 12 h	Tran et al., 2010
	*M. thermophila* laccase expressed in *A. oryzae* (Novozyme)	Free	HBT, SA, VA	100% after 1–8 h	Lloret et al., 2010
	*T. versicolor* (Sigma-Aldrich)	Free	−	95% after 4.5 h	Marco-Urrea et al., 2010b
	*Streptomyces cyaneus*	Free	−	80% after 12 d	Margot et al., 2013a
	*T. versicolor* (Sigma-Aldrich)	Free	−	100% after 2 d	Margot et al., 2013a
	*T. versicolor* (Sigma-Aldrich)	Free	−	90% after 5 h	Margot et al., 2013b
	*C. gallica*	Mesoporous silica spheres	–	70 or 90% at pH 5 (individually or in mixtures); 30% in real wastewater for over 64 reactor volumes	Nair et al., 2013
	*T. versicolor*	Free	HBT	16% after 22 h	Yang et al., 2013b
	*T. versicolor* (Sigma-Aldrich)	Free or combined cross-linked enzyme aggregates of laccase, versatile peroxidase and glucose oxidase	–	100% after 14 h	Touahar et al., 2014
	*Pleurotus florida*	Free	SA	100% after 51 min	Sathishkumar et al., 2014
	*T. versicolor*	Free	HBT, SA	>80% after 24 h	Nguyen et al., 2014a,d
	*M. thermophila* laccase expressed in *A. oryzae* (Novozyme)	Enzymatic membrane reactor	SA	80%	Nguyen et al., 2014c
	*M. thermophila* laccase expressed in *A. oryzae* (Novozyme)	Enzymatic membrane reactor with granular activated carbon addition	SA	80%	Nguyen et al., 2014b
	*M. thermophila* laccase expressed in *A. oryzae* (Novozyme)	Enzymatic membrane reactor	HBT, SA	50% for 72 h (9 hydraulic retention time)	Nguyen et al., 2015
	*T. versicolor* (commercial)	Polyvinyl alcohol/chitosan/multi-walled carbon nanotubes composite nanofibrous membrane	–	100% after 6 h	Xu et al., 2015
	*M. thermophila* laccase expressed in *A. oryzae* (Novozyme)	On granular activated carbon, continuous flow packed-bed reactor	–	100% for 8000 BV	Nguyen et al., 2016a
	*M. thermophila* laccase expressed in *A. oryzae* (Novozyme)	Enzymatic membrane reactor	HBT	60% for 72 h (9 hydraulic retention time)	Nguyen et al., 2016b
	*Phoma* sp. UHH 5-1-03	Free	SA	100% after 22 h	Hofmann and Schlosser, 2016
	*T. versicolor* (Fluka)	Magnetic cross-linked enzyme aggregates	–	85% after 6 h; 95% after 12 h	Kumar and Cabana, 2016
	*Yersinia enterocolitica* laccase expressed in *E. coli*	Free	–	100% after 24 h	Singh et al., 2016
	*T. versicolor* (Sigma-Aldrich)	Cross-linked carbon nanotubes-based biocatalytic membranes	–	94% after 48 h	Ji et al., 2016a
	*P. sanguineus* CS43	Free	–	50% after 8 h	Rodríguez-Delgado et al., 2016
Carbamazepine (anticonvulsant)	*T. versicolor*	Free	HBT	60% after 48 h (repeated treatment every 8 h)	Hata et al., 2010
	*T. versicolor, T. versicolor* (Sigma-Aldrich)	Free	–	37.5% after 12 h	Tran et al., 2010
	*M. thermophila* laccase expressed in *A. oryzae* (Novozyme)	Enzymatic membrane reactor with granular activated carbon addition	SA	70%	Nguyen et al., 2014b
	*T. versicolor* (Sigma-Aldrich)	Hybrid bioreactor of cross-linked laccase aggregates and polysulfone hollow fiber microfilter membrane	–	93% after 72 h	Ba et al., 2014b
	*M. thermophila* laccase expressed in *A. oryzae* (Novozyme)	Enzyme membrane reactor	HBT, SA	60% for 72 h (9 hydraulic retention time)	Nguyen et al., 2015, 2016b
	*T. versicolor* (Sigma-Aldrich)	Biocatalytic TiO2 particle suspension membrane hybrid reactor	p-coumaric acid	71% after 96 h	Ji et al., 2016b
	*T. versicolor* (Sigma-Aldrich)	Cross-linked carbon nanotubes-based biocatalytic membrane	–	60% after 48 h	Ji et al., 2016a
	*M. thermophila* laccase expressed in *A. oryzae* (Novozyme)	On granular activated carbon, continuous flow packed-bed reactor	–	100% for 5,000 BV	Nguyen et al., 2016a
Triclosan (biocide)	*Ganoderma lucidum*	Free	HBT, SA	90% after 24 h	Murugesan et al., 2010
	*T. versicolor* (Sigma-Aldrich)	Chitosan conjugation	–	100% after 6 h	Cabana et al., 2011
	*C. unicolor*	Control porosity carrier silica beads	–	60% after 60 min	Songulashvili et al., 2012
	*T. versicolor* (Sigma-Aldrich)	Free	–	90% after 140 min	Margot et al., 2013b
	*T. versicolor*	Free	HBT, SA	>80% after 24 h	Nguyen et al., 2014a,d
	*C. unicolor*	Mesoporous silica beads	–	100% for 23 h	Debaste et al., 2014
	*P. sanguineus*	Free	–	95% after 8 h	Ramírez-Cavazos et al., 2014
	White-rot fungi (commercial)	Vinyl-modified poly(acrylic acid)/SiO2 mesoporous nanofibers	–	65% after 2 h; 92% after 24 h	Xu et al., 2014
	*Tetracystis aeria*	Free	ABTS	100% after 6 h	Otto et al., 2015
	*P. sanguineus*	Free	–	90% after 5.5 h	Garcia-Morales et al., 2015
	*M. thermophila* laccase expressed in *A. oryzae* (Novozyme)	Enzymatic membrane reactor	–	100% for 72 h (9 hydraulic retention time)	Nguyen et al., 2015, 2016b
	*T. versicolor* (Sigma-Aldrich)	Free	–	83.8% after 5 h	Melo et al., 2016
	*P. ostreatus* (Sigma-Aldrich)	Free	–	0.5757 h^−1^	Sun et al., 2016
	*T. versicolor* (Sigma-Aldrich)	Core-shell magnetic copper alginate beads	HBT	89.6% after 8 h	Le et al., 2016
	*Phoma* sp. UHH 5-1-03	Free	SA	90% after 22 h	Hofmann and Schlosser, 2016

*When multiple mediators were used, only the most efficient one(s) are shown. SA, syringaldehyde; VA, violuric acid*.

In addition to removal of pharmaceutical compounds with laccases, laccase-producing fungi, bacteria and actinomycetes are also evaluated. Laccase is at least partially responsible for pollutant degradation (Marco-Urrea et al., 2010b; Tran et al., 2010; Nguyen et al., 2014d; Popa et al., 2014; Boonnorat et al., 2016; Hofmann and Schlosser, 2016; Vasiliadou et al., 2016). On the contrary, other studies failed to established dependence of pharmaceutical removal on extracellular laccase (Marco-Urrea et al., 2009; Jelic et al., 2012; Yang et al., 2013b). Laccase is also used in combination with other enzymes, such as versatile peroxidase and glucose oxidase (Touahar et al., 2014) or tyrosinase (Ba et al., 2014a) in pharmaceutical removal. Other processes, such as adsorption, are also found to improve compound removal when used in combination with laccase biodegradation (Ba et al., 2014b; Nguyen et al., 2014b,d; Xu et al., 2014; Ji et al., 2016a). Horseradish peroxidase is more efficient than laccase in triclosan removal (Melo et al., 2016), and versatile peroxidase has a wider removal spectrum than laccase (Touahar et al., 2014).

Simultaneous laccase degradation of multiple trace organic contaminants demonstrates that phenolic compounds are generally more easily degraded than non-phenolic compounds (Nguyen et al., 2014a,d), which is expected since phenolic compounds are considered natural laccase substrates. Transformation rates may be different if the compounds are present in solutions of single compounds or in mixtures. For example, Margot et al. found that diclofenac removal is enhanced whereas bisphenol A (BPA) and mefenamic acid elimination is decreased in mixtures (Margot et al., 2013b). Nair et al. also found improved degradation of diclofenac in the presence of BPA and 17-α-ethinylestradiol, whereas degradation of the latter two compounds is not affected in the mixture (Nair et al., 2013). Phenolic compounds such as BPA can serve as a mediator for non-phenolic compounds or may facilitate polymerization (Margot et al., 2013b; Nair et al., 2013; Touahar et al., 2014; Ji et al., 2016a). The majority of work on contaminant removal was carried out in buffers; biodegradation efficiency decreases in real wastewaters (Nair et al., 2013; Touahar et al., 2014; Garcia-Morales et al., 2015; Le et al., 2016), which presents a challenge for laccase applications. Real wastewater has elevated pH compared with the optimized buffer system and potential laccase inhibitors (e.g., organic matter, heavy metals, and ions). However, a few studies still achieved efficient pharmaceutical degradation in real wastewaters (Garcia et al., 2011; Ba et al., 2014a,b; Rodríguez-Delgado et al., 2016).

Laccase mediators HBT and SA are most often used in degradation of PPCPs. Degradation improvement upon mediator addition is significant in the enzymatic membrane reactor and limited in batch incubation (Nguyen et al., 2015). Different conversion mechanisms of triclosan, namely oligomerization in the presence of a laccase mediator and bond cleavage followed by dechlorination in the absence of a laccase mediator have been demonstrated (Murugesan et al., 2010). Laccase-mediated triclosan oligomerization has been confirmed, but dechlorination with only laccase has also been reported (Cabana et al., 2011). More studies comparing transformation pathways with or without a mediator should be carried out. Although, laccase most often has detoxifying effects, laccase and mediator pure preparations and mixture are toxic to bioluminescent bacteria (Nguyen et al., 2016b). Furthermore, inclusion of the natural mediator SA, but not synthetic mediator HBT, in laccase treatment of trace organic contaminants elevates effluent toxicity even though the target contaminants showed negligible toxicity at the low concentration (Nguyen et al., 2014a,c, 2016b).

## Conclusions

Since the first discovery of laccase over 100 years ago, much has been elucidated about the occurrence, biochemistry, sequences, production, and application potentials of this diverse class of enzymes. We have briefly reviewed some recent developments in laccase research with a focus on production and applications in pharmaceutical degradation. Despite the exciting promise laccases bring, applied research is still mostly performed on the laboratory scale. Emphasis should be placed on augmenting the yields and efficiency and lowering application costs, which constitute bottlenecks in scalable and sustainable applications of laccases. While laccase mediators are widely used to aid laccase-catalyzed oxidation, the transformation metabolites as well as their toxicity should always be analyzed. It is clear that more work needs to be done to realize the full potential of these versatile enzymes.

## Author contributions

JY, TN, JL, and XY wrote the manuscript. JY, WL, and XD compiled the tables.

### Conflict of interest statement

The authors declare that the research was conducted in the absence of any commercial or financial relationships that could be construed as a potential conflict of interest.
